# Obstructive sleep apnea and severity of coronary artery disease

**DOI:** 10.22088/cjim.9.3.276

**Published:** 2018

**Authors:** Ali Vasheghani-Farahani, Fatemeh Kazemnejad, Khosro Sadeghniiat-Haghighi, Soleil Saadat, Parya Tavakolipoor, Tahereh Yazdani, Mohammad Alidoosti, Vahid Ghasem-Amooeian, Haleh Ashraf

**Affiliations:** 1 Cardiac Primary Prevention Research Center (CPPRC), Tehran Heart Center, Tehran University of Medical Sciences Tehran, Iran; 2 Students' Scientific Research Center (SSRC), Cardiac primary prevention Research Center, Tehran University of Medical Sciences, Tehran, Iran; 3 Baharloo Hospital, Tehran University of Medical Sciences, Tehran, Iran; 4 Sina trauma research center, Tehran University of Medical Sciences, Tehran, Iran; 5 Obesity Research Center, Sina University Hospital, Tehran University of Medical Sciences, Tehran, Iran

**Keywords:** Atherosclerosis, Coronary angiography, Coronary artery disease, Gensini score, Obstructive sleep apnea

## Abstract

**Background:**

It has been implicated that obstructive sleep apnea (OSA) is associated with increased risk of cardiovascular disease including stroke, myocardial infarction, coronary artery disease (CAD) and hypertension. The aim of this study was to investigate the correlation between OSA and severity of atherosclerosis assessed by angiography.

**Methods:**

This study included 337 patients undergoing diagnostic coronary angiography at Tehran Heart Center, Iran. The Gensini score was obtained from each patient for coronary angiogram, and OSA were assessed by using Multivariable apnea prediction (MAP) risk index on the day of cardiac catheterization. The Gensini scores increased in accordance with increases in the MAP value.

**Results:**

The prevalence rates of three-vessel disease were 68.0% in OSA group and 32.0% in non-OSA group. The MAP index was the most significant independent determinant for the Gensini score.

**Conclusions:**

The independent association between OSA and CAD, even after adjustment for traditional confounders, suggests that, OSA should be taken into account when considering risk factors for CAD.

Obstructive sleep apnea (OSA), a disorder characterized by snoring and repetitive episodes of cessation of breathing during sleep and daytime somnolence, is a prevalent condition that affects approximately 13% and 6% of men and women aged 30-70, respectively (1, 2). About 69% of patients with CAD have OSA (3). OSA is defined as an apnea/hypopnea index (AHI) of more than 5 events/hour. It has been implicated that obstructive sleep apnea is associated with increased risk of cardiovascular disease including stroke, myocardial infarction, CAD and hypertension (4-7). To the best of our knowledge, the correlation between OSA and CAD has not been angiographically confirmed. Studies have emphasized on the important role of diagnosis and treatment of OSA in patients with CAD (7, 8). Continuous positive airway pressure (CPAP) ,the gold standard therapy for OSA, has been shown to reduce cardiovascular mortality and improve cardiovascular risk factors including blood pressure, lipid profile and insulin sensitivity (9-12). The Iranian population is becoming aged and more obese, two conditions that increase the risk of OSA. We aimed to assess the relationship between severity of OSA and coronary atherosclerosis by application of coronary artery angiography, Gensini scoring evaluation system, and other risk factors which may contribute to CAD.

## Methods

***Subjects: ***This study included 337 patients undergoing diagnostic coronary angiography at Tehran Heart Center, Iran. Exclusion criteria were a prior history of revascularization or oxygen therapy, chronic obstructive pulmonary disease, daytime hypoxemia and the regular use of hypnotic agents. Informed consent was obtained from all patients. The protocol was approved by the Ethics Committee of Tehran University of Medical Sciences, and the study was conducted in conformity with the current revision of the Declaration of Helsinki. 

***Instruments: ***The patients' clinical and demographic data were gathered by a semi-structured interview, while the interviewer was blind to the patient's angiographic results. Height, weight and BMI were measured. A family history of premature CAD was determined as cardiovascular event in <45 years old female or <55 years old male 1st-degree relative. Venous blood samples were obtained after at least 10 hours of overnight fasting.

***Angiographic estimation of coronary atherosclerosis: ***Coronary angiography was performed by the femoral approach and included at least 5 views of the left coronary artery and 2 views of the right coronary artery. The Gensini score which determines the severity of stenosis (13) was calculated based on coronary angiogram, and the left ventricular ejection fraction was determined from the left ventriculogram. The Gensini score was computed by assigning a severity score to each coronary stenosis according to the degree of luminal narrowing and its geographic importance. Lumen diameter reduction of 25%, 50%, 75%, 90%, 99% and total occlusion are graded as 1, 2, 4, 8, 16 and 32, respectively. This score is then multiplied by a factor that takes into account the importance of the lesion’s position in which the more proximal it I,s the higher weighting score it receives. The Gensini score was expressed as the sum of the scores for all the coronary arteries. The subjects were divided into 4 groups according to the Gensini score: group A or negative result (Gensini score = 0), group B or nominal result (Gensini score ≤ 5), group C (Gensini score: 6 - 30) and group D or advanced CAD (Gensini score > 30). Patients with one or more stenosis less than 50% were defined as having minimal CAD. 

***Estimation of obstructive sleep apnea: ***OSA was assessed by using multivariable apnea prediction (MAP) risk index on the day of cardiac catheterization. Multiple logistic regression models were used to incorporate BMI, age and gender into a MAP index. We accepted a threshold MAP index ≥ 0.50 as diagnostic of OSA. Thus, the patients were divided into 2 groups, according to the severity of OSA: OSA group (0.50≤ MAP value) and non- OSA group (MAP value < 0.50).

***Definition of the metabolic abnormalities: ***The metabolic syndrome (MetS) was defined as the presence of three or more of the following criteria: fasting plasma glucose ≥110 mg/dl or previous diagnosis of diabetes, HDL-C < 40 mg/dl and 50 mg/dl in men and women, respectively, triglycerides ≥150 mg/dl, blood pressure ≥130/85mmHg or the use of antihypertensive drug therapy and overweight[14]. Since data on waist circumference were not available, overweight is defined as BMI>25 kg/m^2^. 

**Statistical analysis: **Data were analyzed using SPSS for windows statistical package Version 17. Student's t test was used to compare continuous variables and chi-square test was used to compare categorical variables across the two groups. The correlations between variables were studied with the Pearson test. Multiple linear regression analysis was used to assess the relationship between MAP index and Gensini score adjusted for the effects of age, gender, family history of CAD, smoking or opium addiction status, hypertension, diabetes, hyperlipidemia, BMI or the presence of MetS. Multiple, stepwise, linear regression analysis also was performed to identify which variables best explained the variance in the Gensini score. A p-value of < 0.15 was first used to identify candidate variables, and then removed variables from the regression model if p value was < 0.1. A p<0.05 was considered significant.

## Results

337 patients (169 men, 168 women) with the mean age of 57±11 years were included. Sleep studies revealed OSA in 119 (35.3%) patients with a MAP value of 0.50 and higher. Clinical characteristics and serum profiles of patients in the two study groups are summarized in table 1. Normal coronary angiography was found in 19.3%, nominal angiographic findings in 7.4%, and positive angiographic findings in 73.3%. Subjects with OSA were older and SBP, serum levels of triglycerides and total cholesterol and BMI was significantly higher and the level of HDL-C was lower in this group. There were no significant differences regarding DBP, fasting plasma glucose, family history of ischemic heart disease, smoking or opium addiction history between two groups (table 1). 

**Table 1:** Clinical and metabolic characteristics of the study subjects with and without sleep apnea

**Characteristics**	**Positive sleep apnea study** **MAP index ≥ 0.50**	**Negative sleep apnea study** **MAP index < 0.50**	**Pvalue**
Patients, No.	119	218	
Age, year	59.9±10. 4	55.5±11.1	<0.001
Gender			
Male	87 (73.1%)	82 (37.6%)	<0.001
Female	32 (26.9%)	136 (62.4%)	
Body mass index (kg/m^2^)	30. 5 ± 6.6	26.6 ± 3.7	<0.001
Smoking	11 (9.2%)	32 (14.7%)	0.174
Opium addiction	1 (0.8%)	5 (2.3%)	0.430
Family history	17 (14.3%)	49 (22.5%)	0.085
Systolic blood pressure, mmHg	139.7 ± 20.3	134.2 ± 18.4	0.012
Diastolic blood pressure, mmHg	83.2 ± 12.5	82.1 ± 12.1	0.420
Subjects with hypertension	71(59.7 %)	108 (49.5 %)	0.048
Triglycerides, mg/dL	208.1 ± 121.1	168.5 ± 99.3	0.003
Total cholesterol, mg/dL	186.2 ± 51.3	171.2 ± 52.8	0.012
HDL-cholesterol, mg/dL	38.7 ± 9.8	43.8 ±11.5	<0.001
Dyslipidemia	102 (85.7%)	163 (74.8%)	0.026
Fasting blood glucose, mg/dL	118.4 ± 39.9	116.7 ± 69.1	0.811
Hyperglycemia	32 (26.9%)	46 (21.1%)	0.280
Subjects with at least three of the following: hypertension, hyperglycemia, overweight high triglyceride and low HDL-cholesterol values	80 (67.2%)	106 (48.6%)	0.001

Data are presented as the means ± SD or as numbers and percentages. HDL;high-density lipoprotein, MAP; multivariable apnea risk.

**Figure 1:**
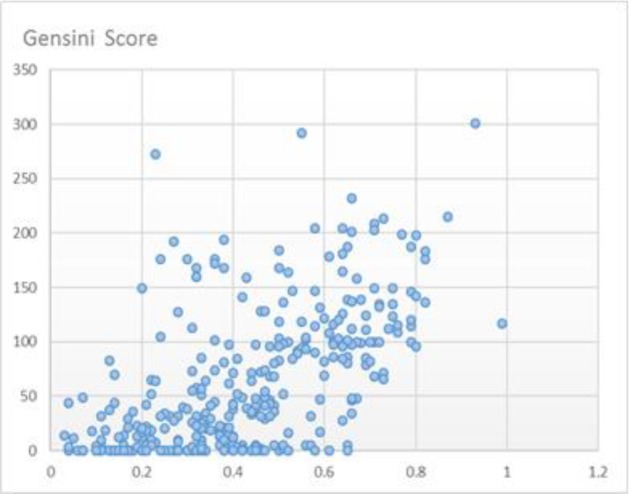
The Gensini scores increased in accordance with increases in the Multivariable Apnea risk index value

**Figure 2:**
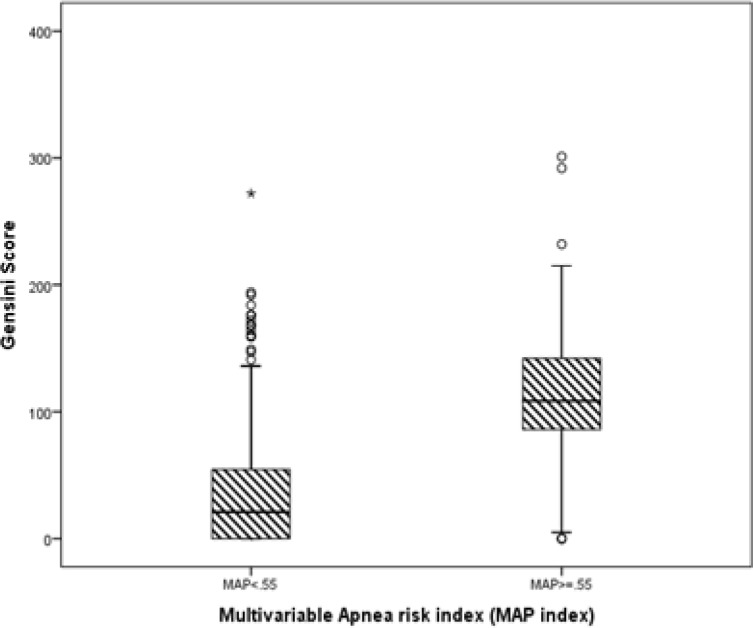
The Gensini scores in apnea group was significantly higher than those in non-apnea group

The Gensini scores increased in accordance with increases in the MAP value, and the scores in OSA group were significantly higher than those in non-OSA group (figures 1 and 2). 

Table 2 shows a comparison of coronary angiography and left ventriculography data across the MAP value quartiles. The prevalence of three-vessel disease was 68.0% in OSA group and 32.0% in non-OSA group. The proportion of patients with OSA also increased across the four CAD severity groupings (table 2). MAP value and coronary angiography data progressively worsened in subgroups with progressive increases of BMI (data not shown). Although MAP index is used mostly as dichotomous variable for the qualitative estimation of OSA, there was a linear trend effect of association with MetS as the severity of OSA increased in terms of MAP. A significant relationship between a higher MAP index with the increase in the number of features of MetS in the whole study group was also demonstrated (P<0.05). Univariate analysis showed that MAP was significantly associated with Gensini score. The association of Gensini score with MAP index both unadjusted and adjusted for the effect of other risk factors is displayed in table 3.

**Table 2:** A Comparison of coronary arteriography and left ventriculography data across the levels of sleep apnea severity

**Variable**	**Positive sleep apnea study** **MAP index ≥ 0.50**	**Negative sleep apnea study** **MAP index < 0.50**
**Diseased vessels, No**.		
Minimal CAD	33 (27.7)	141 (64.7)
Single vessel disease	46 (38.7)	49 (22.5)
Two vessels disease	22 (18.5)	19 (8.7)
Three vessels disease	17 (14.3)	8 (3.7)
Left main leision	1 (0.8)	1 (0.5)
**Gensini score**		
Total score	109.55 ± 60.49	33.58 ± 47.28
Score = 0	6 (5)	59 (27.1)
Score ≤ 5	5 (4.2)	20 (9.2)
Score: 6 – 30	3 (2.5)	51 (23.4)
Score > 30	105 (88.2)	88 (40.4)
Ejection fraction, %	46.07 ± 13.32	50.44 ± 12.45

**Table 3:** Association of sleep apnea alone or with control variablesiIntroduced with a measure of coronary artery severity (Gensini score)

	**Model 1** a		**Model 2** b		**Model 3** c	
	**Univariate (r)**	**P value**	**Multivariate (ß)**	**P value**	**Multivariate (ß)**	**P value**
Age, yr	0.208	<0.001	0.148	0.001	0.138	0.002
Male (0 = no, 1 = yes)	0.344	<0.001	0.282	<0.001	0.268	<0.001
Body mass index (kg/m2)	0.256	<0.001	0.191	<0.001	0.192	<0.001
Smoking (0 = no, 1 = yes)	0.046	0.399	-0.020	0.689		
Opium addiction (0 = no, 1 = yes)	0.201	<0.001	0.190	<0.001	0.187	<0.001
Family history (0 = no, 1 = yes)	0.050	0.363	0.093	0.032	0.090	0.031
Systolic blood pressure	0.078	0.153	-0.046	0.396		
Diastolic blood pressure	0.032	0.561	-0.007	0.894		
Total cholesterol	0.093	0.089	0.003	0.945		
Triglycerides	0.138	0.011	0.01	0.845		
HDL-cholesterol	-0.155	0.004	0.04	0.41		
Fasting blood glucose	0.073	0.181	0.051	0.234		
Metabolic syndrome	0.197	<0.00	0.121	0.026	0.103	0.021
Sleep apnea, MAP index	0.596	<0.001	0.377	<0.001	0.376	<0.001

a Model 1: unadjusted estimate of Gensini score.

b Model 2: adjusted for age, gender, family history, smoking or addiction status, hypertension, diabetes, hyperlipidemia, BMI or the presence of MetS.

c Model 3: Multiple, stepwise, linear regression to identify best variables explaining the variance in the Gensini score.

## Discussion

In the present study, we have investigated the OSA estimated by MAP index as a correlation of severity of coronary artery disease verified by Gensini score. We found the Gensini score is significantly higher in patients with an increased risk of apnea. In addition, the proportion of patients with OSA increased with severity of CAD, from 19.8%% among the negative angiographic subjects or minimal CAD to 68.0% among the patients with three vessel disease. Furthermore, although, the prevalence and components of MetS including high blood pressure, high fasting blood glucose level and dyslipidemia increase while the severity of OSA increases, multiple regression analysis of coronary risk factors showed that the OSA verified by MAP index was the most significant independent determinant of the severity of coronary atherosclerosis. A growing bulk of evidence from studies has been suggesting that OSA is an independent risk factor for CAD (7). 

The result of our study in an Iranian sample was similar to the research studies of Gan et al.(15), Hayashi et al. (16) and Inami et al. (17) which revealed a positive correlation between Gensini score and the severity of OSA. Hayashi et al. (16) examined the relation between nocturnal oxygen desaturation index (ODI) and the Gensini score in patients with CAD diagnosed by coronary angiography. Multiple regression analysis showed that the ODI was the most significant, independent determinant of the Gensini score among the coronary risk factors tested. There is growing experimental and clinical evidence for an independent association of OSA with the development and/or severity of MetS. On the contrary, MetS and its components—in particular, obesity, hypertension and insulin resistance—may have conductive influence on the development of OSA. The connection between obesity and OSA may be mutually perpetuating.

 The exact mechanisms by which obesity may increase the risk of OSA or vice versa are still obscure. The detrimental effects of fat deposition on upper airway anatomy and its function, and leptin resistance have been suggested (18-21). Considerable overlap between OSA and obesity makes it difficult to identify the role each plays in many cardiovascular diseases. The association between OSA and insulin resistance, a putative background of the MetS, has been investigated in a number of reports (22, 23). The severity of OSA may affect insulin resistance to a greater extent in nonobese patients with OSA, indicating that OSA *per se *may be associated with insulin resistance, although concomitant obesity or visceral fat accumulation are predominant risk factors for insulin resistance (24, 25). The increasing recognition and acceptance of a link between OSA and MetS prompts the question of whether OSA occurs as part of the fundamental pathophysiology of MetS, or OSA, via repetitive nocturnal hypoxemia, systemic inflammation, and other mechanisms, promotes the components of MetS. 

Therefore studies on the association between OSA and cardiovascular sequelae need to be controlled for these important potential confounders. In our study, the percentage of patients presenting at least two metabolic abnormalities was significantly higher in the OSA group. OSA patients with MetS had higher degrees of Gensini score. As discussed in detail above, the impact of potential confounders was carefully controlled in the present study. The strength of the association between OSA and CAD severity was decreased by about 18% via introducing the confounding factors, but remained significant and MAP index was the most significant independent determinant for the Gensini score. Assuming OSA precedes CAD, the relationship of OSA and CAD may be explained by several mechanisms including hemodynamic changes (brady tachycardia, hypoxi chypertensive episodes) and sympathetic hyperactivity which may in turn evoke endothelial damage via mechanical strain, vasospasm and thrombogenesis (26-29). Also, repetitive hypoxia cause oxidative stress which then results in lipid peroxidation, generation of inflammatory agents and all these factors can promote the development of atherosclerosis (18, 30-33). 

 Some limitations need to be considered when interpreting the results of this study. We did not have access to measurements of waist circumference. Hence, the prevalence of MetS might be higher than what we found in this study. Because of the design of our study, we cannot establish causality. The diagnosis of OSA in our study was made based on MAP index.

 Although MAP index has been extensively used in different investigations to assess OSA with a good sensitivity and specificity (34), polysomnographic study was not performed to confirm the diagnosis. In addition, the results of this study cannot be extrapolated to other ethnic groups. Undoubtedly, well- designed prospective crossover controlled studies with larger samples and longer follow-up and rigorous characterization of subjects would be required to further elucidate the link between OSA and CAD, as well as the associated mechanisms. 

In conclusion these data suggest a high prevalence of OSA in CAD patients. The independent association between OSA and CAD, even after adjustment for traditional confounders, suggests that great attention should be paid to the screening of OSA when considering the risk factors for CAD, as OSA can easily be diagnosed, and in many cases represents a readily treatable condition. The high prevalence of OSA and the morbidity and mortality thought to be associated with it, have resulted in a view that OSA may be as big a public health hazard as smoking and purchasers are increasingly being urged to support sleep services. 
